# Elliptical Fourier analysis of hominoid radius shape: implications for *Ardipithecus ramidus*

**DOI:** 10.1242/bio.061938

**Published:** 2025-06-03

**Authors:** Isabella Araiza

**Affiliations:** Department of Anthropology, University of Washington, 4218 Memorial Way Northeast, Seattle, OR 98105, USA

**Keywords:** Australopithecus, Locomotion, Hominin evolution, Knuckle-walking

## Abstract

The evolution of bipedalism in the hominin lineage remains a controversial topic. The recovery of skeletal material from Aramis, the Middle Awash Project study area in Middle Awash, Afar Regional State, Ethiopia, has the potential to elucidate the transition to terrestrial bipedalism. The 4.4-million-year-old hominin *Ardipithecus ramidus* (ARA-VP-6/500) is represented by a relatively complete skeleton, including a complete radius. Its describers argued that it lacked features associated with suspensory behaviors, vertical climbing, and knuckle-walking. To test this hypothesis, I collected a comparative sample of radii comprising of *Homo sapiens* (*n*=27), six species of extant apes (*n*=96), two species of cercopithecoids (*n*=31), and two fossil hominins, and quantified whole bone shape using elliptical Fourier analysis (EFA). Dorsal radial morphology effectively partitions taxa by size and locomotion. The radii of knuckle-walking chimpanzees, and particularly gorillas, retain robust epiphyses and high degrees of lateral curvature, in contrast to other species. The robusticity and unique, directional curvature observed in the African ape radius may be related to knuckle-walking. The radius of ARA-VP-6/500 exhibits distinct characteristics among hominins, falling exclusively within gorilla morphospace. Although *Ar. ramidus* postcrania were proposed to lack features indicative of an ancestry involving knuckle-walking, vertical climbing, and suspensory behavior, this study instead contributes to growing lines of evidence suggesting that humans likely evolved from a knuckle-walking ancestor.

## Teaser

**Summary:** The postcranial morphology of *Ardipithecus* has been argued to lack African ape-like features. Instead, dorsal radial morphology suggests the robusticity observed in the ARA-VP-6/500 radius reflects its unique evolutionary histories.

## INTRODUCTION

The 4.4-million-year-old hominin *Ardipithecus ramidus* preserves a partial skeleton (ARA-VP-6/500) and portions of at least 16 other individuals (White et al., 1994; 2009). Originally, this hominin was recognized as a new species of *Australopithecus* and stem hominin to *Au. afarensis* (White et al., 1994). However, a taxonomic revision issued this hominin to a new genus *Ardipithecus*, consisting of only two known members (*Ar. ramidus* and *Ar. kadabba*) of the hominin clade. The holotype for *Ar. ramidus* is represented by ARA-VP-6/1, a set upper and lower teeth (upper left I^1^, C, P^3^, P^4^, right I^1^, C, P^4^, M^2^, lower right P_3_, and P_4_; White et al., 1994; 1995) discovered in Aramis, west of the Awash River in the Middle Awash study area, Afar depression, Ethiopia, in 1993. Additional sources of evidence for this taxon include a partial cranial base, mandibular fragments and long bones. Its locomotor status was estimated from the anteriorly placed foramen magnum (White et al., 1994), suggesting this creature was likely bipedal. It was distinguished from *Australopithecus* by its smaller postcanines, thinner molar enamel, and small, sexually dimorphic canines similar to *Homo* (White et al., 1994; Suwa et al., 2021). In 2009, the postcranial skeleton, ARA-VP-6/500, was described in greater detail, but was very fragmented with some post-depositional deformation (Lovejoy et al., 2009a,b,c,d; White et al., 2009). The estimated body mass for this individual was 51 kg, compared to Lucy with an estimated body mass of 30-35 kg. (White et al., 2009). The postcranial skeleton has undergone intense virtual reconstructions, and it was determined from these morphologies that *Ar. ramidus* was likely a facultative biped that is probably ancestral to *Australopithecus* (White at al., 1994; White et al., 2009). The hominin status for ARA-VP-6/500 was distinguished by its derived characteristics of the skull, pelvis, and foot that would have contributed to balance and support during bipedal walking (Lovejoy et al., 2009a; Suwa et al., 2009; White et al., 2009, 2015). The skeletal material was interpreted to lack any features consistent with suspensory or knuckle-walking behaviors (Lovejoy, 2009). Thus, ARA-VP-6/500 retains a mosaic of primitive and derived features throughout its skeleton that are described as an unknown early form of bipedalism involving careful arboreal clambering capabilities with positional orthogrady and pronogrady (Lovejoy et al., 2009a,b; White et al., 2009, 2015; Simpson et al., 2019).

Much of the ARA-VP-6/500 right radius is complete, although damaged distally of the radial tuberosity. The radius shows greater distal articular surface angulation relative to the shaft axis not found in early hominins, consistent with a more laterally facing radial facet on the scaphoid (Lovejoy et al., 2009a). Additionally, the *Ardipithecus* radius has a more medially facing radial tuberosity, a trait that is commonly found in knuckle-walkers (Aiello et al., 1990; Hunt, 2016). The radius is not described in detail; however, the radius/tibia ratio is 0.95, similar to generalized arboreal quadrupeds like macaques and *Proconsul*, indicative of an adaptation to ‘careful climbing’ (Lovejoy et al., 2009b; White et al., 2009). Its estimated brachial index is similar to *Australopithecus afarensis* (A.L. 288-1) and falls within the range of *Pan* (Lovejoy et al., 2009c). It should be noted that some non-suspensory early Miocene apes also overlap with *Pan* on the brachial index, while humans and *Gorilla* overlap as well (see Fig. S3 in Lovejoy et al., 2009c). Additionally, Prang et al. (2021) found that *Ardipithecus* likely descended from a suspensory, *Pan*-like ancestor instead of a generalized monkey-like ancestor based on shared hand morphology with chimpanzees and bonobos. Inferences on the upper limb were solely derived from the ulna. The authors describe it as a generalized bone with an elbow joint for full extension, but lacks any suspensory features (Lovejoy et al., 2009a,b,c,d). Based on the descriptions the authors noted above, they argue that *Ardipithecus* provides evidence that the last common ancestor (LCA) of hominins and panins was a generalized, large-bodied African ape that did not evolve the specializations observed in chimpanzees and gorillas.

In addition to the ARA-VP-6/500 radius, this project will also investigate the 1.98-million-year-old partial skeleton from Malapa, South Africa, *Australopithecus sediba* (MH2) represented by the U.W. 88-85 complete right radius (Berger et al., 2010; Dirks et al., 2010; Pickering et al., 2011). Radial morphology consists of notable longitudinal curvature, a somewhat circular, lateral positioned head with a thinner neck, a medially placed radial tuberosity, and a distal margin that is projecting mediolaterally (Churchill et al., 2018). *Au. sediba* shares distal radial morphology with other australopiths, but the dorsal ridges on the radiocarpal articular margins are distinct (Kimbel and Delezene, 2009). The skeleton exhibits features associated with climbing and suspension as in suspensory apes in greater capacity than *Au. afarensis* (Churchill et al., 2013; Rein et al., 2015, 2017; Churchill et al., 2018; Meyer et al., 2023) but also bipedal features used in terrestrial and arboreal locomotion (Zipfel et al., 2011; Schmid et al., 2013; DeSilva et al., 2013; Williams et al., 2013; DeSilva et al., 2018; Williams et al., 2018, 2021).

Primates exhibit a range of specializations in locomotion (Napier, 1967; Hunt, 1991b, 2016), yet most primates possess a relatively generalized postcranial skeleton, permitting locomotor versatility in arboreal and terrestrial environments (Le Gros Clark, 1959; Patel, 2010; Elton et al., 2016). The radius is a critical location to study as the transmission of forces occurs here in primates as it contributes to elbow and wrist mobility. Although forelimb morphology varies among primates, the direction and degree of radial curvature may indicate locomotor behaviors. Longitudinal bone curvature has been suggested to enhance bending predictability at the expense of bone structural strength (Lanyon, 1980; Bertram and Biewener, 1988; Jade et al., 2014), while remodeling to a straight bone would maximize mechanical strength (Frost, 1964; Jade et al., 2014). Ground reaction forces influence forelimb function, counteracting the effects of postural behaviors on diaphyseal bowing (Bertram and Biewener, 1992). Long bones *in vivo* are mechanically loaded in response to bending and withstand varying degrees of bone strain (Lanyon and Baggott, 1976; Lanyon et al., 1982; Ruff et al., 2006). Recent studies have shown that radial shape correlates with locomotor behaviors across mammals (Milne, 2016; Henderson et al., 2017; Milne and Granatosky, 2021). In chimpanzees, a bowed forelimb increases the moment arms of the pronators and supinators to aid in rotation of the wrist (Hunt, 2016). Research on upper limb morphology in strepsirrhines has associated mediolateral bending with grasping during feeding (Fabre, et al., 2018), permitting rotation of the forelimb and hand position adjustments. Additionally, research on rat ulnae exposed to mechanical forces exhibited mediolateral thickening of the diaphysis near the midshaft in response to strain (Robling et al., 2002). Given these findings, assessing mediolateral curvature of the dorsal radius could provide valuable insights into hominoid and fossil hominin locomotor behaviors.

This study conducts a comparative analysis of *Ardipithecus* radial morphology and will investigate two hypotheses: (1) radial curvature is reflective of locomotor behaviors in hominoids (i.e. knuckle-walking, suspension, digitigrady, brachiation, bipedalism), with more pronounced curvature associated with knuckle-walking, while straighter radii are indicative of bipedality. (2) *Ar. ramidus* will exhibit morphological affinities to particular living catarrhines. If *Ardipithecus* was a generalized arboreal climber with bipedal traits, its radial morphology should resemble that of more arboreal primates (i.e. *Pongo*, hylobatids). The null hypotheses are that radial curvature does not correlate with locomotor behavior and the *Ar. ramidus* radius occupies a unique shape space among catarrhines.

## RESULTS

**Fig. 1.**
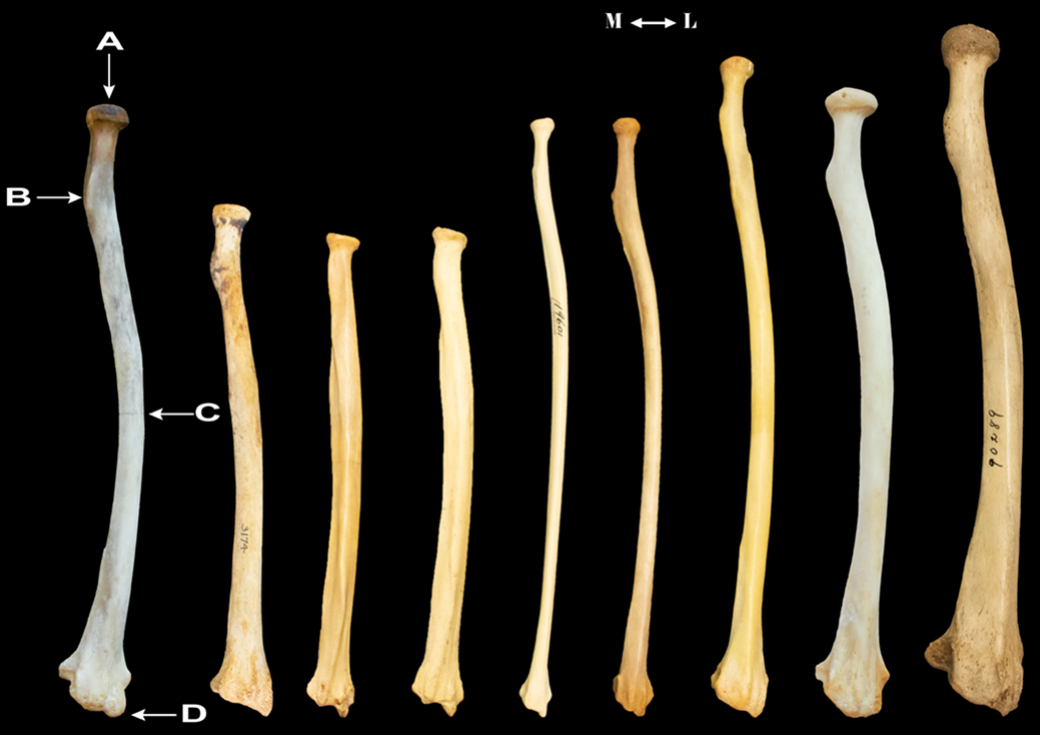
**Qualitative features observed in the primate radius from dorsal view.** (A) radial head, (B) radial tuberosity, (C) midshaft curvature, (D) styloid process. From left to right: *P. paniscus*, *H. sapiens*, *Papio*, *Mandrillus*, *H. lar*, *S. syndactylus*, *P. pygmaeus*, *P. troglodytes*, *G. gorilla*).

**Fig. 2.**
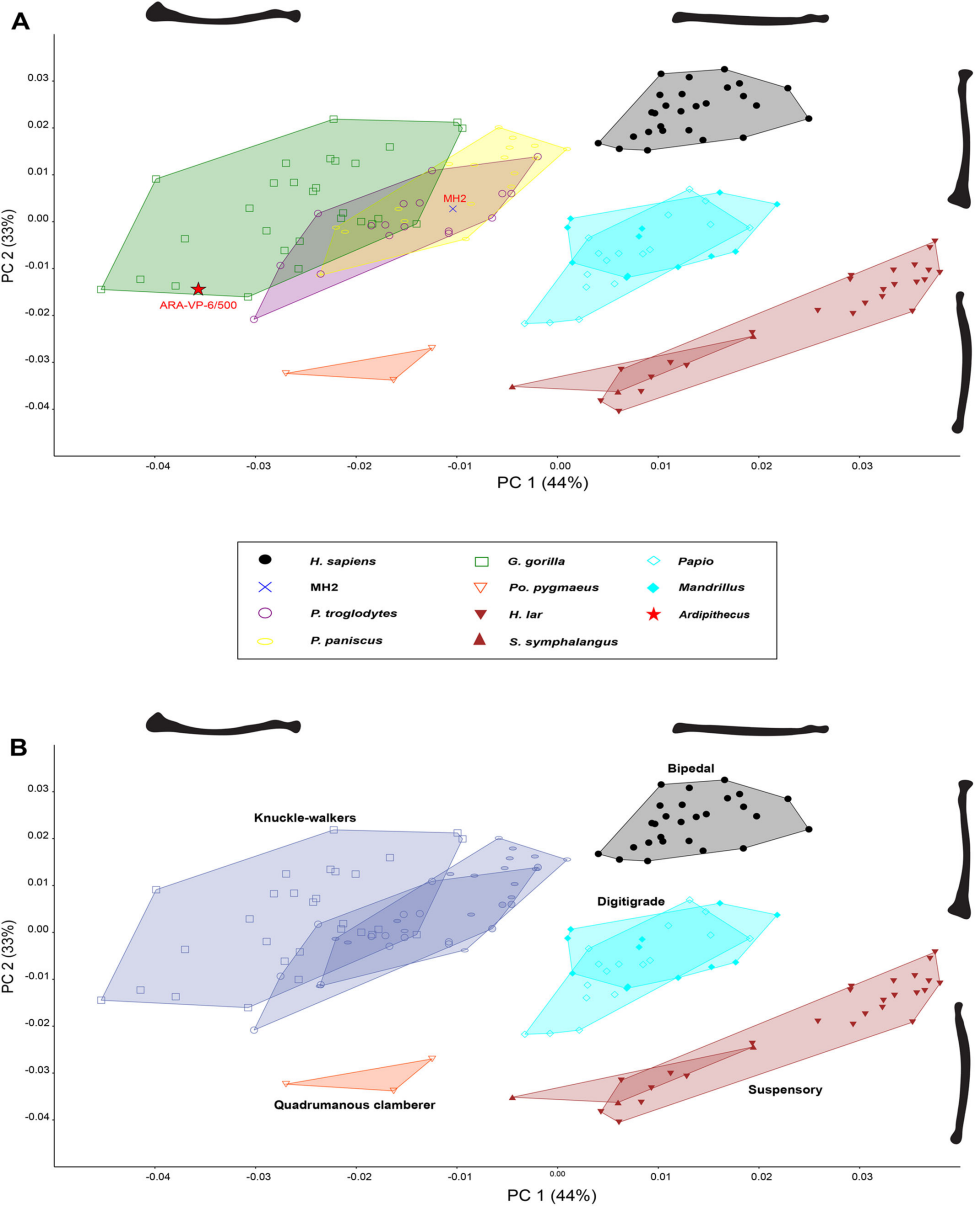
**Biplot of the first two Principal Components from elliptical Fourier descriptors of radius contours in dorsal view.** (A) PC1 (44%) reflects locomotor behaviors with positive values representing brachiating primates and negative values representing knuckle-walking primates. PC2 (33%) reflects shaft curvature and with positive values representing a straighter diaphysis and negative values representing a higher degree of curvature. (B) The biplot categorizes primate taxa into locomotor groups.

**Fig. 3.**
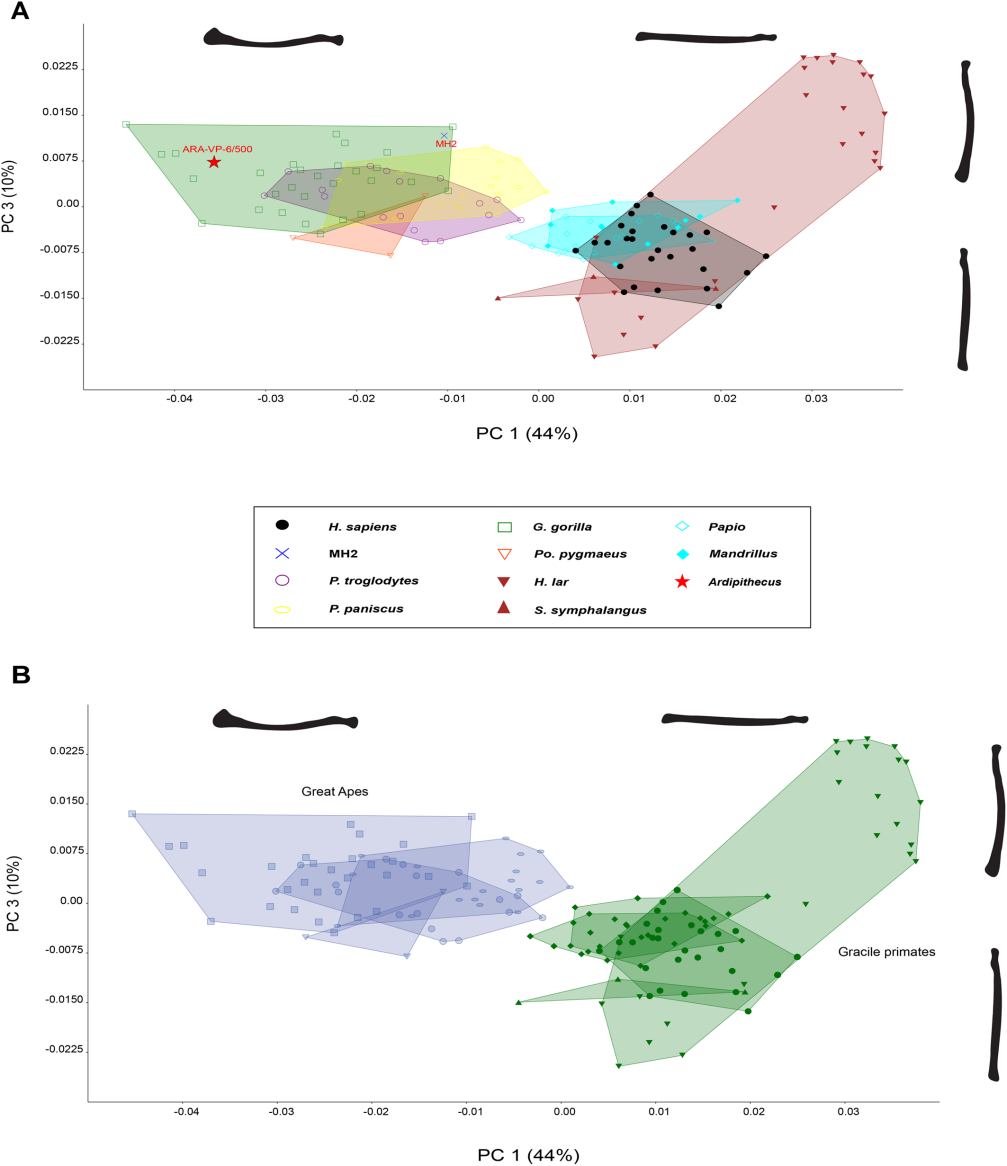
**Biplot of the first and third Principal Components from elliptical Fourier descriptors of radius contours in dorsal view.** (A) PC1 (44%) reflects locomotion and PC3 (10%) reflects quantification of proximal and distal epiphyseal curvature. (B) The biplot distinguishes great apes from all other primates, including anatomically modern humans.

Table 1 summarizes several radial traits commonly discussed in the literature for various primate taxa. The morphological features used to assess robusticity and gracility in the PCA are illustrated in Fig. 1. Principal Component (PC) 1 explains 44% of the variance in the dataset (Figs 2A and 3A), representing overall curvature of the long axis of the radius. Positive values describe a relatively straighter midshaft and negative values represent a higher degree of midshaft curvature.

**Table 1.** **Radial traits present in primates and their associated robusticity classification** (R, robust; G, gracile; I, intermediate)

Feature	*P. paniscus*	*P. troglodytes*	*Papio*	*Mandrillus*	*H. lar*	*S. syndactyulus*	*G. gorilla*	*H. sapiens*	*P. pgymaeus*
Head shape	Elevated **R**	Elevated **R**	Flattened **G**	Flattened **G**	Elevated **R**	Elevated **R**	Elevated **R**	Elevated **R**	Elevated **R**
Tuberosity placement	Medial **R**	Medial **R**	Antero-medial **G**	Antero-medial **G**	Medial **R**	Medial **R**	Medial **R**	Antero-medial **G**	Medial **R**
Mid shaft curvature	Curved **R**	Curved **R**	Curved **R**	Curved **R**	Curved **R**	Curved **R**	Curved **R**	Straight **G**	Curved **R**
Styloid process length	Projecting **R**	Projecting **R**	Projecting **R**	Projecting **R**	Projecting **R**	Projecting **R**	Projecting **R**	Short **G**	Projecting **R**

**Fig. 4.**
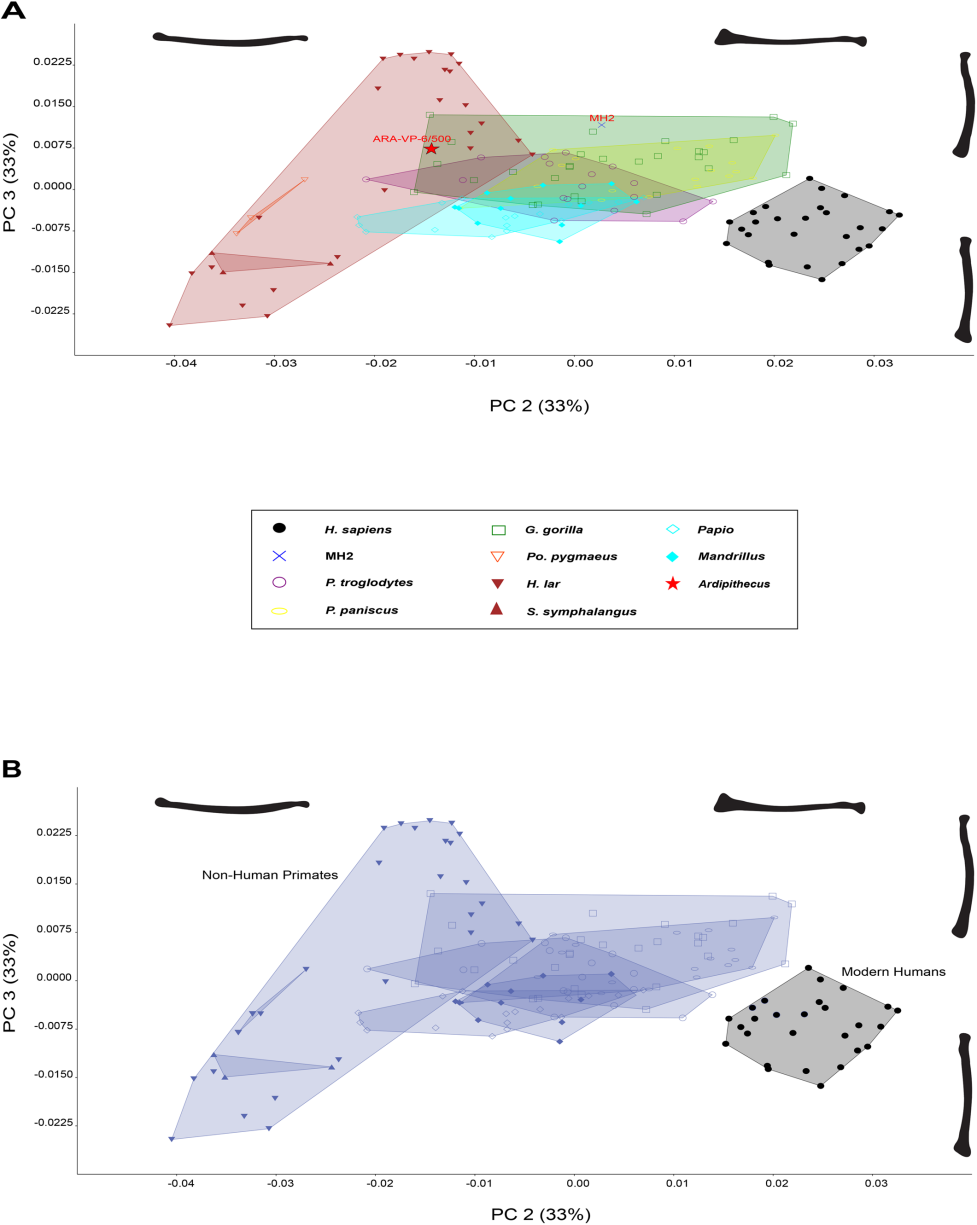
**Biplot of the second and third Principal Components from elliptical Fourier descriptors of radius contours in dorsal view.** (A) PC2 (33%) reflects shaft curvature and with positive values representing a straighter diaphysis and negative values representing a higher degree of curvature. PC3 reflects the quantification of proximal and distal epiphyseal curvature. (B) The biplot demonstrates a clear separation of modern humans from non-human primates.

PC2 explains 33% of variance (Figs 2A and 4A), which reflects distal and proximal radial morphology, with positive values representing a pronounced radial head, larger radial tuberosity, larger distal epiphysis and a prominent styloid process, and negative values representing a smaller radial head, radial tuberosity and distal epiphysis with a receding styloid process. A lateral lip on the radial head is also present in positive values and absent in negative values.

PC3 explains 10% of variance (Figs 3A and 4A), quantifying distal epiphyseal curvature and radial head morphology with positive values reflecting curving distal and proximal epiphyses and negative values representing a straight distal epiphysis.

A biplot of PC1 against PC2 (Fig. 1A) explains 77% of variation (Fig. 2B) and partitions taxonomic groups by locomotor groups (i.e. knuckle-walking, bipedalism, suspension). *G. gorilla*, *P. troglodytes*, *P. paniscus*, and *Pongo* have more curved and robust radii than other anthropoids. Hylobatids occupy a distinct morphospace with less shaft curvature and distal robusticity than other sample taxa, suggesting that high (positive) PC1 values signal brachiation and extremely low (negative) PC2 values (<−0.02) correspond to a more arboreal lifestyle. Cercopithecoids and *H. sapiens* separate into a distinct morphospace intermediate of African apes and hylobatids. This position within the PCA reflects slight shaft curvature and moderate robusticity of the distal radius in comparison to *Pongo*, hylobatids, and African apes. The human and cercopithecoid sample have intermediate positive values relative to great apes and hylobatids, except two cercopithecoid individuals that exhibit low PC1 values. MH2 falls directly within the distribution of *Pan*, and ARA-VP 6/500 falls on the outer margin of the *Gorilla* morphospace.

Within a biplot of PC1 against PC3 (Fig. 3A), 54% of variation is explained as great apes separate from lesser apes, cercopithecoids, and humans (Fig. 3B). Great apes tend to exhibit greater radial curvature and robusticity with a larger distal epiphysis while gracile primates retain less overall curvature and a smaller distal epiphysis. Here, both hominins fall within the great ape morphospace, more specifically within that of *Gorilla*. The importance of this biplot demonstrates the separation of great apes from gracile primates (i.e. hylobatids, papionins, and *H. sapiens*) in the shape space.

A biplot of PC2 and PC3 (Fig. 4A) explains 43% of variation showing most primates clustering closely together, with hylobatids stretching from positive to negative PC3 values. There is a high degree of overlap in the nonhuman primate sample along all axes. The majority of gorillas and bonobos, as well as some chimpanzees, fall on the positive axis of PC2 and have a thicker distal radius compared to hylobatids and *Pongo* and a prominent radial head comparable to humans, while negative PC2 values retain smaller epiphyses and a receding styloid process. *Pongo*, hylobatids and the majority of cercopithecoids occupy a large morphospace with most specimens having low PC values. This biplot importantly demonstrates humans separate from all other taxa (Fig. 4B) displaying a straighter radial shaft, wider distal epiphysis, receding styloid process, and unique radial head not found in other non-human primates. The hominin fossils also fall within the non-human primate morphospace with MH2 positioned within *Gorilla* and intermediate in shape space compared to ARA-VP-6/500 lying on the outer margins of *Gorilla* and hylobatids.

## DISCUSSION

Using elliptical Fourier techniques, the dorsal radius was quantified to understand how mediolateral curvature and varying levels of robusticity signals distinct locomotor behaviors in primates. Results showed that each taxonomic group partitioned into a distinct morphospace, reflecting differences in locomotor behaviors and forelimb loading patterns among primates (Fig. 4B). Humans, hylobatids, and cercopithecoids exhibit a straighter, less robust radius than the great apes. The human forelimb is incredibly unique and clearly separates from other primates, likely due to the liberation of our forelimbs from a supportive function. The adaptation of bipedalism allows the forelimb to instead act as a pendulum, counteracting vertical displacement produced by the lower limbs during walking (Umberger, 2008; Collins et al., 2009; Pontzer et al., 2009). This added stability of locomotion helps decrease the energetic cost involved in walking, whereas a restriction to arm swing increases energetic costs (Anderson and Pandy, 2001; Herr and Popovic, 2008; Umberger, 2008; Collins et al., 2009; Meyns et al., 2013). However, humans retain a more robust distal epiphysis similar to some African apes, possibly indicative of an evolutionary history of weight-bearing (Jenkins and Fleagle, 1975), as knuckle-walkers experience increased compressive loads, requiring greater subchondral bone density (Carlson and Patel, 2006). This supports the idea that forelimb load distribution plays a significant role as an adaptive response in the primate skeleton, leading to curvature or bowing of the radius. African apes, particularly gorillas, heavily utilize their forelimbs for weight-bearing, unlike other taxa. Chimpanzees and gorillas possess powerful supinator and pronator muscles, increasing the moment arms of a curved radius to allow powerful rotation of the wrist (Stern and Larson, 2001; Hunt, 2016), while maintaining a fully extended elbow (Simpson et al., 2018; Milne and Granatosky, 2021). The differences found in the primate radial tuberosity are likely reflecting position rather than size as African apes retain a more medially positioned radial tuberosity to increase the action of the biceps brachii in supination (Aiello et al., 1990). Hylobatids predominantly engage in more arboreal and suspensory behaviors, experiencing minimal weight-bearing on their forelimbs yet still possess powerful supinator, pronator muscles, and elbow flexors due to rapid forearm rotation during brachiation (Rose, 1988; Vanhoof et al., 2020). Because suspensory primates use their forelimbs to free hang from substrates, their forelimbs are subjected to increased tensile loads and less compressive loads, effectively decreasing subchondral bone density (Carlson and Patel, 2006). Although *Pongo* exhibits arboreal tendencies, orangutans also employ various hand positions when climbing and fist walking in terrestrial environments (Thorpe and Crompton, 2006; Oishi et al., 2009; Thorpe et al., 2009). Orangutans are quite similar to gibbons in forelimb musculature; however, the forelimb is subjected to compressive loads like African apes while clambering in arboreal environments (Cant, 1987; Hunt et al., 1996; Thorpe and Crompton, 2006), leading to radial curvature similar in chimpanzees and gorillas. Papionins are adapted to terrestrial habitats and engage in digitigrady, facilitating more load distribution on the hind limbs to enhance forelimb mobility (Patel and Wunderlich, 2010; Druelle et al., 2017). The forelimb is restricted to movement with an asymmetrical, flattened radial head and large ulnar styloid process that limit supination and instead provides more stability and less rotation at the wrist compared to hominoids (O'Conner and Rarey, 1979; Rose, 1988; Hunt, 2016). Rather, monkeys possess powerful elbow extensor and digital flexor muscles to support quadrupedal walking, running, and leaping (Hunt, 2016). These findings support the hypothesis that radial mediolateral curvature reflects locomotor behaviors and functional adaptations in primates.

Fossil hominins occupy different shape spaces in the PCA results. Previous research indicates the ulnar morphology of *Au. sediba* resembles that of other bipeds (Araiza et al., 2021; Meyer et al., 2023), with evidence of increased arboreality (Rein et al., 2017). Rein et al. (2017) identified a suspensory and climbing signal in the MH2 ulna, which might explain the shared morphospace with African apes. In a biplot of PC1 against PC2, MH2 falls within the morphospace of *Pan* and the knuckle-walking distribution. This hominin has greater longitudinal curvature, a more medially positioned radial tuberosity, a lateral lip present on the radial head, and a larger distal epiphysis with a projecting styloid process. In a biplot of PC1 against PC3, *Au. sediba* falls out of any known morphospace, but near the great ape distribution with ARA-VP-6/500 distinguishing this hominin from the gracile primate group (Fig. 2B). The biplot of PC2 against PC3 separates this individual from the more robust features in the ARA-VP-6/500 radius. Consequently, a discriminant function analysis classified MH2 into the knuckle-walker locomotor group (Table S3).

Dorsal radial morphology of ARA-VP 6/500 (*Ar. ramidus*) falls within a morphospace exclusively occupied by *Gorilla*. In a biplot of PC1 against PC2, the overall radial curvature of ARA-VP-6/500 is greater than the majority of primates included in this sample. This hominin has similar distal mediolateral robusticity as the most robust gorillas, but a more curved distal epiphysis than MH2. *Ardipithecus* retains a less pronounced radial head with a lateral lip, a medially placed radial tuberosity, and projecting styloid process. In a biplot of PC1 against PC3, *Ardipithecus* is within the *Gorilla* morphospace and great ape distribution, further demonstrating the morphological similarities between the two species.

Lastly, a biplot of PC2 and PC3 place *Ar. ramidus* within the non-human primate distribution alongside *Au. sediba*, exhibiting a strongly bowed radius. A discriminant analysis was performed on ARA-VP-6/500 and was classified to the knuckle-walker locomotor group (Table S3).

Although ARA-VP-6/500 and MH2 fall within the knuckle-walking distribution, their postcranial morphology suggests that they did not engage in this behavior. Features such as an anteriorly placed foramen magnum, a shortened upper pelvis compared to great apes, a broad, sagittally facing iliac blade, an elongate lumbar region, and the terrestrial propulsive role of the lateral rays of the foot, suggest *Ar. ramidus* was a biped that did not knuckle-walk (Suwa et al., 2009; White et al., 2009; Lovejoy et al., 2009c). Similarly, MH2 shares several bipedal characteristics, including a valgus knee and human-like ankle, a bicondylar angle, a mobile lower back with a curved lumbar region, and a highly derived os coxa similar to *Homo* (Berger et al., 2010; Williams et al., 2021). Instead, the position of MH2 and ARA-VP-6/500 are likely indicative of their evolutionary histories, that is, the position of ARA-VP-6/500 within the *Gorilla* morphospace and MH2 in the *Pan* morphospace, reflects a largely primitive radius shape inherited from a knuckle-walking ancestry. As the forelimbs of *Ardipithecus* were largely freed from terrestrial locomotion and it was not a tool-user, stabilizing selection for climbing and suspensory behavior likely remained in place. This interpretation does not support prior hypotheses of generalized or monkey-like morphologies and positional behaviors of *Ardipithecus* (Lovejoy, 2009; Lovejoy et al., 2009a; White et al., 2009) and instead suggests that the locomotor repertoire of the LCA likely included terrestrial plantigrady and suspensory adaptations, common to extant African apes (Williams, 2012; Prang, 2019; Prang et al., 2021; Williams et al., 2023).

Overall, the results of this study show that, the dorsal radial morphology of *Ar. ramidus* contradicts the initial interpretation that ARA-VP 6/500 lacked specializations of African apes and shares generalized morphometric affinities with early Miocene hominoids. Thus, the presence of an African ape-like radius in ARA-VP-6/500 provides additional support for the hypothesis that the LCA was a knuckle-walker, contrary to assertions made by Lovejoy et al. (2009a,b,c,d) and White et al. (2009, 2015). Based on the current evidence, *Ar. ramidus* is best interpreted as representing an early habitual biped whose radial morphology is a relic of its evolutionary history and also facilitated climbing and suspensory behavior. Similarly, *Au. sediba* was a habitual biped that retained adaptations to arboreal behavior.

### Conclusion

The results obtained from the elliptical Fourier analyses of the radius in dorsal view supports its reliability for quantifying radius shape. This approach effectively distinguishes between different locomotor groups among apes, demonstrating the utility of EFA in elucidating functional adaptations of the radius, particularly in the context of locomotor behavior in primates. Consequently, this methodology shows potential in deducing forelimb function and locomotor patterns in fossil taxa in future studies.

This study finds support for the hypothesis that radial morphology appears to signal locomotor behaviors and functional adaptations in primates. Moreover, variation in radial curvature corresponds to locomotor-related differences, emphasizing its reliability in primate locomotion studies. While the hominin sample is generally thought to be bipedal, I suggest radial morphology reflects their evolutionary histories and arboreal locomotor behaviors due to the non-use of forelimbs in terrestrial locomotion. Consequently, the hypothesis that *Ar. ramidus* does not retain knuckle-walking or suspensory behaviors must be rejected considering its *Gorilla*-like radial shape. The retention of an African ape-like radius in *Ardipithecus* provides evidence that bipedalism may have evolved from a knuckle-walking ancestor. This hypothesis will be tested by the subsequent study on *Ar. ramidus* fossils and those of other early hominins.

## MATERIALS AND METHODS

The material obtained for this study includes 154 non-pathological adult individuals (Table 2): *Homo sapiens* (*n*=27), *Pan troglodytes* (*n*=17), *Pan paniscus* (*n*=18), *Gorilla gorilla* (*n*=30), *Pongo pygmaeus* (*n*=3), *Hylobates lar* (*n*=25), *Symphalangus syndactylus* (*n*=3), *Mandrillus* (*n*=14), and *Papio* (*n*=17). Specimens are housed in the following museum collections: Museum of Comparative Zoology, Harvard, MA, USA; Royal Museum of Central Africa, Tervuren, Belgium; and the American Museum of Natural History, New York, NY, USA. Right radii were examined to determine if pathologies were present and accordingly discarded from the sample.

**Table 2.** Extant species sample composition of dorsal radii collected from photographs

Species	(*n*)
*Australopithecus sediba*	1
*Ardipithecus ramidus*	1
*Gorilla gorilla*	30
*Homo sapiens*	27
*Hylobates lar*	25
*Mandrillus sp.*	14
*Pan paniscus*	18
*Pan troglodytes*	17
*Papio sp.*	17
*Pongo pygmaeus*	3
*Symphalangus syndactylus*	3
Total	156

Data for fossil hominins were collected from images of *Ardipithecus ramidus* (*n*=1) represented by ARA-VP 6/500 (White et al., 2009) and *Australopithecus sediba* (*n*=1) represented by U.W. 88-85 from the MH2 skeleton (Berger et al., 2010; Churchill et al., 2018). These fossil images were taken from published sources in dorsal view. It is assumed that each specimen from the literature was positioned in its correct anatomic orientation, as variation in orientation would have confounding effects on shape analysis. An additional test was performed by tilting the radius several degrees on its axis laterally and medially in SHAPE on a subsample of modern human individuals (*n*=72). This analysis found no difference among centered or tilted radii and can reliably conclude that tilting radii does not confound these results (see Fig. S1).

Images of extant taxa were collected in dorsal view via photography using a Nikon D5300 camera without flash, with dimensions of 6000×4000 pixels, an exposure time of 1/80 seconds, and a focal length of 20 mm. The camera was positioned pointing vertically down from a height of 75 cm to reduce parallax distortion. Any inaccurately oriented images were excluded to strengthen the techniques used in this paper. Elliptical Fourier analysis (EFA) has been shown to provide an accurate mathematical characterization of shape (Kaesler and Waters, 1972; Kuhl and Giardina, 1982; Persoon and Fu, 1986). EFA is a powerful biometric tool particularly suited to the description of fossils as it can delineate any closed two-dimensional (2D) contour (Crampton, 1995; Schmittbuhl et al., 2007; Athreya, 2009; Caple et al., 2017). Consequently, EFA can quantify the entirety of long bone shafts (Araiza et al., 2021), unlike the subset of selected 2D landmarks used in geometric morphometrics. While recent methods using three-dimensional (3D) landmarks and semi-landmarks provide an alternative approach to capturing long bone curvature, they rely on researcher defined points of interest rather than a continuous representation of shape. Similarly, long bone curvature subtense, a method that quantifies bone curvature from a straight line, has several drawbacks compared to landmark data collection, including a higher degree of measurement error and potential data loss between landmarks (De Groote et al., 2010). Additionally, curvature subtense reduces shape complexity to a single-dimensional measurement, limiting its ability to capture the entire morphology of a bone. To improve upon and avoid the drawbacks of these methods, this study employs elliptical Fourier shape analysis, which allows a more comprehensive shape quantification of the entire bone than a selection of predetermined landmarks.

**Fig. 5.**
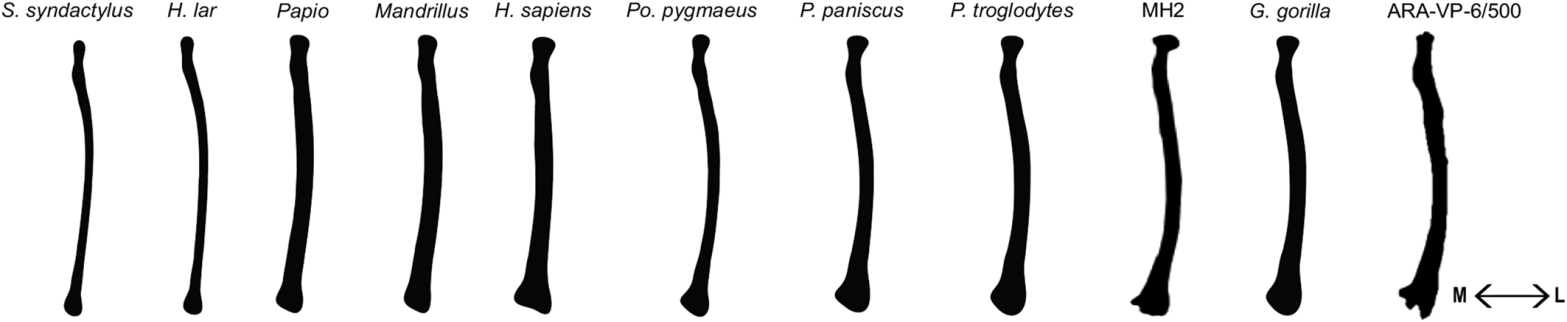
**A representation of the average dorsal radial shape for each primate taxa including fossil hominins**. (From left to right: *Symphalangus syndactylus*, *Hylobates lar*, *Papio* sp., *Mandrillus* sp., *H. sapiens*, *Pongo pygmaeus*, *Pan paniscus*, *Pan troglodytes*, *Au. sediba* (MH2), *Gorilla gorilla*, *Ar. ramidus* (ARA-VP-6/500). These radial profiles originate from the SHAPE program.

2D images of the dorsal aspect of radii were processed within Photoshop and standardized to a 24-bit, 664-pixel width bitmap files. The entire shape of each radius was quantified using EFA techniques (Kaesler and Waters, 1972; Kuhl and Giardina, 1982; Rohlf and Archie, 1984; Persoon and Fu, 1986; Carlo et al., 2011; Caple et al., 2017; Araiza et al., 2021; Meyer et al., 2023). EFA converts the closed outline of an object into a chain code or coordinate-based representation for computational analysis. Parameters used on the chain code involve maximum harmonic number and normalization method. Each harmonic corresponds as a frequency that describes shape details of an outline, represented mathematically by sine and cosine waves. The elliptical Fourier descriptors (EFDs) are these coefficients of waves, encoding shape information. These coefficients transform geometric data from spatial to frequential domains around the outline of a shape. Chain codes are converted into EFDs without relying on landmarks, making it suitable for analyzing complex shapes (Carlo et al., 2011). The first harmonic captures the basic outline of the shape, while additional harmonics produce finer details, increasing the accuracy of shape reconstruction. Multiple harmonics affect size, shape, and orientation, allowing complex contours to be reconstructed from EFDs. This process permits the profile perimeter of an object to be described using ellipses as shown in Fig. 5 (Kaesler and Waters, 1972; Rohlf and Archie, 1984; Carlo et al., 2011; Caple et al., 2017). Shape was assessed using the suite of SHAPE programs (Iwata, 2002) to generate four EFDs per harmonic (20). The files were manually normalized based on the longest radius using the program CHC2NEF within the SHAPE package. This approach allows a substantial volume of shape data to be collected compared to techniques relying on sparsely positioned landmarks, thereby avoiding the omission of information between these landmarks. To test repeatability of this analysis, interobserver differences on a small subsample of *Pan* (*n*=2) and *Papio* (*n*=8) dorsal radii were collected (Fig. S2). These primates were chosen due to their extreme morphological differences including varying degrees of robusticity. A biplot of PC1-PC2 were collected by two separate observers and reveals a negligible degree of inter-observer variability, highlighting the reliability and replicability of this methodology.

Principal Component analysis (PCA) was performed on the coefficients of the EFDs to summarize variation (Rohlf and Archie, 1984; Carlo et al., 2011) using PAST software. Elliptical Fourier analysis detects distinct morphological variability and produces accurate visual representations of objects. Principal Components derived from EFDs allow for statistical interpretation of variation present within the data by reducing the dimensionality of data. Thus, EFDs enable Principal Components to statistically interpret variations present within the data.

This study uses the following locomotor groups to distinguish the role of entire radial morphology with various locomotor behaviors: knuckle-walking (*Gorilla* and *Pan*), bipedal (*Homo*), suspensory (*Hylobates* and *Symphalangus*), quadrumanous clamber (*Pongo*), and digitigrade (*Papio* and *Mandrillus*). These classifications broadly align with other prior research regarding locomotor groups (Napier, 1967; Spoor et al., 2007). This paper acknowledges that non-human primates engage in a diverse array of locomotion and using broad categories obscures their behavioral patterns (Hunt et al., 1996), however this permits the analysis to partition the sample into plausible locomotor groups discussed within paleoanthropological literature. Additionally, it is worth considering that primate taxa are known to engage in multiple forms of locomotion and positional behaviors that researchers often disagree on how these categories are defined (Keith, 1902; Hunt et al., 1996; Thorpe and Crompton, 2006).

Hylobatids (*Hylobates* and *Symphalangus*) are defined as true brachiators, predominately suspending themselves along arboreal substrates or brachiating through forest canopy quickly (Fleagle, 1976, 1980; Remis, 1995; Doran and Hunt, 1994; Hunt, 2016). Consequently, Hylobatids are categorized into the suspensory locomotor group. Although *Pongo* and hylobatids both engage in suspensory behaviors, *Pongo* more frequently engages in quadrumanous clambering and terrestrial fist walking (Loken et al., 2013, 2015; Ancrenaz et al., 2014; Hunt, 2016), whereas hylobatids engage in increased bouts of brachiation (Thorpe and Crompton, 2006). Thus, *Pongo* is placed into the quadrumanous clamber locomotor category.

*Pan* engages in suspensory behaviors but to a lesser extent than their more arboreal relatives and instead engage in higher frequencies of terrestrial behaviors (Hunt, 1991a,b; Doran, 1993a,b; Videan and McGrew, 2002; D'Août et al., 2004; Hunt, 2016). Nearly all suspensory behaviors occur during food gathering on small arboreal supports; however, these behaviors vary by population, social factors, age, and food availability (Susman et al., 1980; Hunt, 2016). *Pan* is also observed to engage in vertical climbing when entering a feeding tree, often utilizing smaller supports to avoid fatigue (Hunt, 1992a,b; Hunt, 2016). Yet considerable amount of time is spent on the ground when resting or travelling between feeding trees (Hunt, 1992a,b). Hence, *Pan* is placed with the knuckle-walking group.

*Gorilla* shares some similarities to *Pan* in that gorillas engage in suspensory behaviors and vertical climbing; however, the occurrence of suspension decreases during ontogeny as body size increases quickly (Doran, 1997). Adult gorillas engage mostly in knuckle-walking; however, this amount varies by subspecies, sex, age, and region (Doran and Hunt, 1994; Doran, 1996; Doran, 1997; Saurringhaus et al., 2022). This study incorporates Western gorillas (*G. gorilla*), which are more arboreal compared to other gorilla subspecies spending up to 10% of their time in the trees (Sarringhaus et al., 2022). However, the *Gorilla* sample have been categorized into the knuckle-walking group due to their predominate terrestrial behaviors, which are documented in the literature.

### Limitations of this study

This study relies on the assumption that the images taken from the literature of fossil radii are in correct anatomical position. While elliptical Fourier descriptors prove to be an effective tool when accounting for allometry from form and function of the radius, it is important to note that the approach presented here does not aim to replace comprehensive morphological analyses. Therefore, we urge readers to exercise caution, as the strength of our findings did not consider the length of the radius, which has been deemed useful in distinguishing primate locomotor patterns. It is important to note that this study is limited to observations from dorsal view, and we did not account for various metric and nonmetric characteristics identified in other views of the radius in apes. The ARA-VP-6/500 radius is mostly complete yet damaged, further affecting the reliability of these results. Additionally, we did not consider the functional elbow joint complex that includes the ulna and humerus, nor did we examine relative linear sizes of structures like the distal radius or radial head, or indices related to radius length, which provide additional biomechanical insights into forelimb function (Preuschoft and Demes, 1985; Vizcaíno and Milne, 2002). Although our results provide evidence of an African ape-like radius in ARA-VP 6/500 and MH2, it is important to consider that the *Gorilla/Pan*-like morphology observed in these fossils could potentially be attributed to homoplasy, indicating similar traits evolving independently due to unidentified functional factors. Additionally, we caution that the locomotor groups do not encompass variations related to environmental factors, sex, ontogeny, or population differences within taxa, which could potentially yield statistically distinct outcomes. While this study offers compelling data regarding locomotion, these hypotheses require further investigation with more fossil taxa and primate samples.

## Supplementary Material

10.1242/biolopen.061938_sup1 Supplementary information
