# Supplementary material for: Elliptical Fourier analysis of hominoid radius shape: implications for Ardipithecus ramidus
Source: Biol Open. 2025 Jun 3;14(5):bio061938. doi: 10.1242/bio.061938 (PMC12171094; doi:10.1242/bio.061938)
Supplement: Supplementary information [file biolopen-14-061938-s1.pdf]

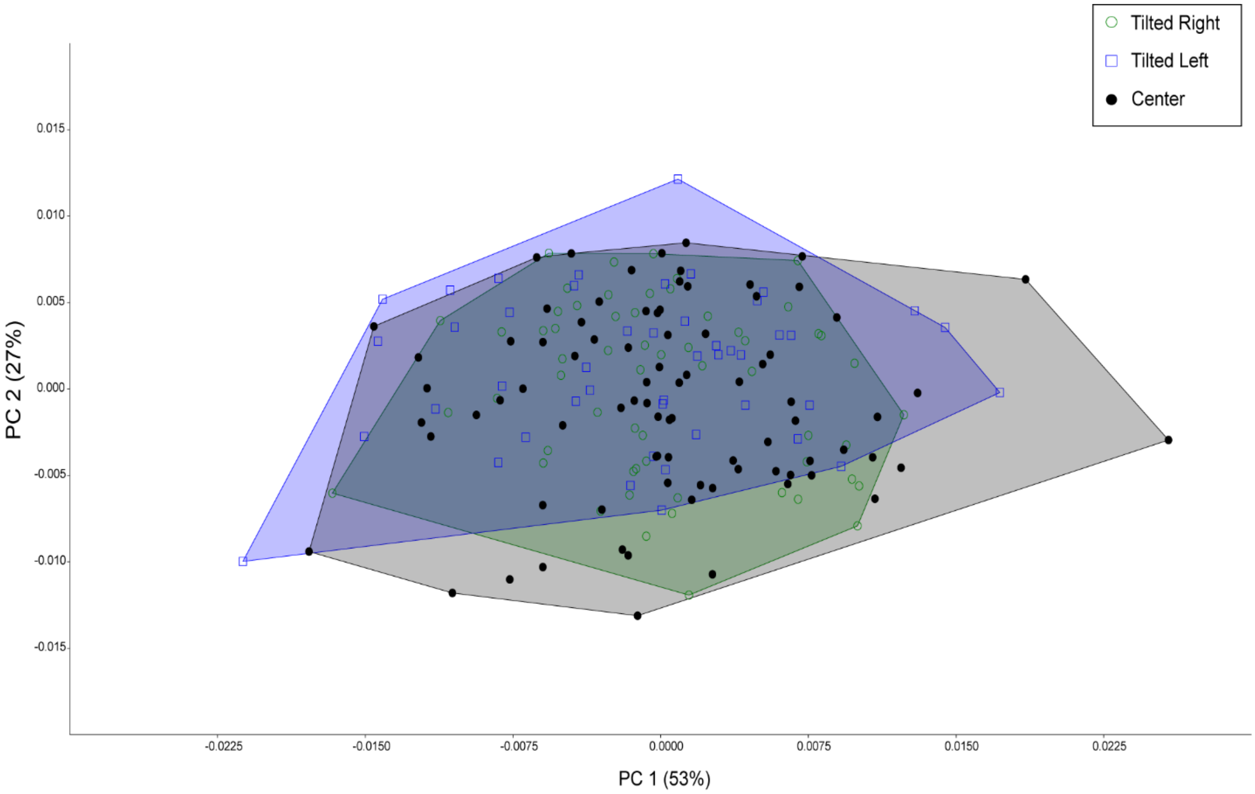

Fig. S1. To test for erroneous positioning of the radius in dorsal view, a subsample of modern human individuals (n = 72) were collected from 3D models. The radii were tilted by five degrees laterally and medially on its axis within Meshlab and screen captured to perform an EFA in SHAPE. This analysis found little to no difference among centered or tilted radii and can reliably conclude that tilting radii does not confound these results.

Biology Open • Supplementary information

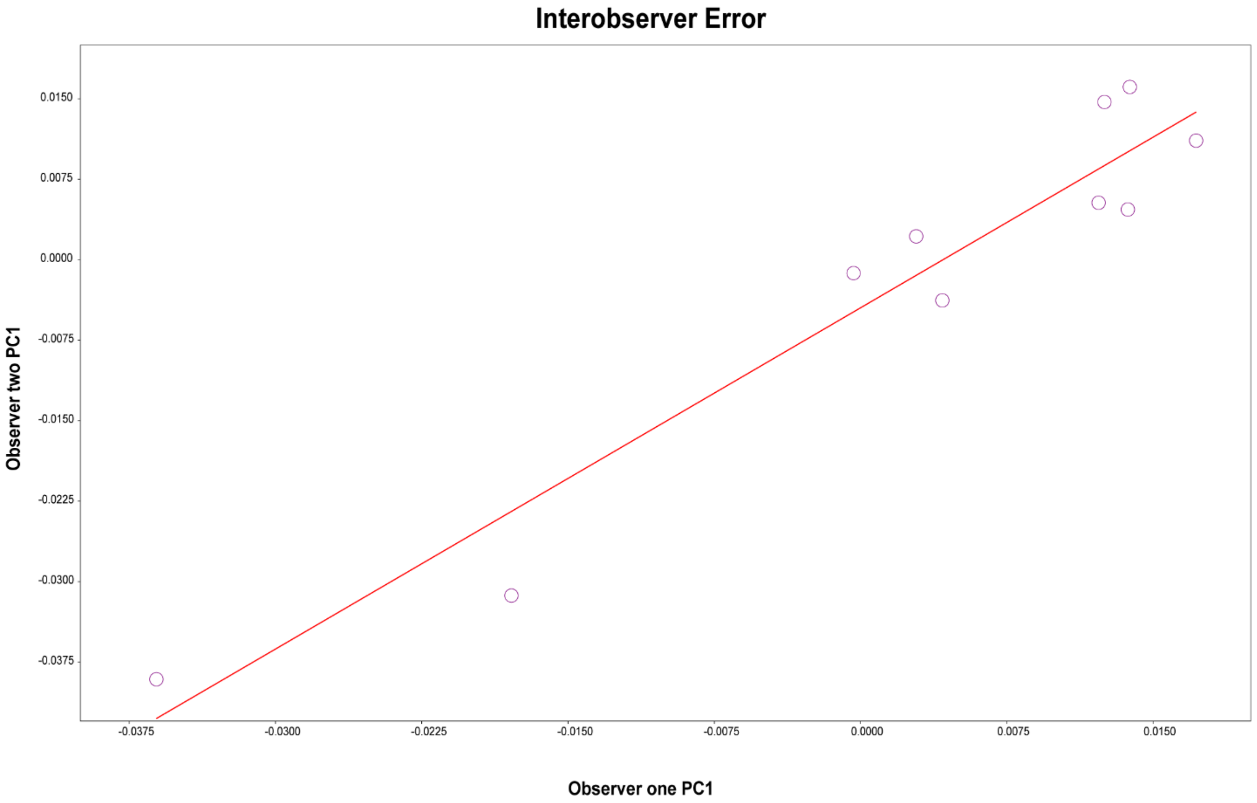

Fig. S2. Interobserver differences in data collected from a subsample of *Pan* and *Papio* dorsal radii (n = 10). Ordinary Least Squares regression of PC1 values recorded by observer 1 (I.F.X.A.) and observer 2 (M.R.M.). Residual sum of squares (SS) = 0.00023, residual mean square (MS) = 2.9E-05, total SS = 0.003, F = 100.25, p = 0.0001.

Biology Open • Supplementary information

Biology Open (2025): doi:10.1242/bio.061938: Supplementary information

Table S1. A Multivariate analysis of variance with Bonferroni-corrected pairwise comparisons on PC1-PC3. The results of a MANOVA conducted in PAST 4.17 indicated most taxa were significantly distinct from one another. Yet there was not a significant difference found between *P. paniscus* and *P. troglodytes* (p = 5.7), *Papio* and *Mandrillus* (p = 12.6), *S. syndactylus* and *Pongo* (p = 5.1), or *S. syndactylus* and *H. lar* (p = 1.6).

**Species**	*Po. Pygmaeus*	*P. troglodytes*	*P. paniscus*	*G. gorilla*	*H. lar*	*Mandrillus*	*Papio*	*H. sapiens*	*S. syndactylus*
Po. Pygmaeus		0.00070397	8.19E-05	2.21E-09	1.63E-07	0.067763	0.04616<br>4	2.09E-08	5.0838
P. troglodytes			5.7388	1.40E-07	4.39E-26	3.30E-10	4.72E-12	1.16E-12	2.43E-07
P. paniscus				4.15 -07	2.10E-27	4.33E-11	4.08E-13		
G. gorilla					1.98E-40	4.50E-21	6.26E-24	4.74E-24	1.91E-14
H. lar						1.93E-14	1.09E-15	1.24E-32	1.5609
Mandrillus							12.638	4.22E-13	0.0092331
Papio								2.89E-15	0.0071681
H. sapiens									1.30E-10
S. syndactylus									

Biology Open • Supplementary information

Table S2. Confusion Matrix

A confusion matrix was generated from a discriminant function analysis (CVA) to compare species classification against their true identification. While the majority of the sample was correctly classified (jackknife = 71%), some misclassifications occurred among overlapping species pairs (i.e., *P. troglodytes* and *P. paniscus*, *H. lar* and *S. syndactylus*, *Papio* and *Mandrillus*.

Species	*H. sapiens*	*P. paniscus*	*P. troglodytes*	*G. gorilla*	*Po. Pygmaeus*	*S. syndactylus*	*H. lar*	*Mandrillus*	*Papio*	***Total***
H. sapiens	27	0	0	0	0	0	0	0	0	0
P. paniscus	0	12	6	0	0	0	0	0	0	18
P. troglodytes	0	5	11	1	0	0	0	0	0	17
G. gorilla	0	4	22	0	0	0	0	0	0	30
Po. pygmaeus	0	0	0	0	3	0	0	0	0	3
S. syndactylus	0	0	0	0	0	2	1	0	0	3
H. lar	0	0	0	0	0	11	14	0	0	25
Mandrillus	0	0	0	0	1	0	0	8	5	14
Papio	0	0	0	0	1	0	0	6	10	17
Total	27	21	21	23	5	13	15	14	15	154

Table S3. A discriminant function analysis (CVA) was performed on the two hominin fossils to further confirm their classification status in PAST. MH2 and ARA-VP-6/500 are correctly classified into the knuckle-walker locomotor group as shown in the original analysis (jackknife = 71%).

**Fossil Hominin**	**Given group**	**Classification**	**Jackknife**
ARA-VP-6/500	?	*G. gorilla*	71%
UW 88-85	?	*P. troglodytes*	71%

Biology Open • Supplementary information
